# Distribution of Coal Power, Pollution, and Poverty

**DOI:** 10.1001/jamanetworkopen.2026.42335

**Published:** 2026-11-05

**Authors:** Eleanna M. Melcher, William R. Buckingham, Sarah A. Keller, Amy J. H. Kind

**Affiliations:** 1 Department of Population Health Sciences, University of Wisconsin School of Medicine and Public Health, Madison; 2 Center for Health Disparities Research, University of Wisconsin School of Medicine and Public Health UW Hospital and Clinics, Madison; 3 Department of Medicine, University of Wisconsin School of Medicine and Public Health, Madison

## Abstract

**Importance:**

The adverse social exposome—life-course exposure to harmful social-environmental conditions—is associated with poor health outcomes. Exposure to heavy metals in air pollution from coal-fired power plants (CPP) may represent an underlying mechanism of this disparity, yet the association between the US adverse social exposome measured with the Area Deprivation Index (ADI) and CPP remains unknown.

**Objective:**

To characterize the populations and adverse social exposome (indexed by ADI) of communities neighboring operational CPP.

**Design, Setting, and Participants:**

Cross-sectional study of the US population using data from the 2023 Neighborhood Atlas. Data were analyzed from March to April 2026.

**Exposure:**

Proximity to CPP was operationalized using 4 circular buffer zones: 3, 10, 25, and 50 miles in radius.

**Main Outcomes and Measures:**

(1) Characterize each buffer zone by ADI and (2) determine the potential population-level exposure to CPP. Census block group delineations and population characteristics are from time-concordant US Census and American Community Survey data.

**Results:**

The 2023 US population included 335 563 144 people (169 517 541 [50.5%] female, 66 294 296 [19.8%] Hispanic, 269 268 846 [80.2%] not Hispanic or Latino, and 212 002 396 [63.1%] White). A total of 2 783 004 people (0.83% of the US population) lived within 3 miles of a CPP. This group was more likely to live in the highest ADI neighborhoods as compared with the lowest ADI neighborhoods. Compared with the US population, these individuals were more likely to be American Indian and Alaska Native, White, and non-Hispanic. Populations living in the lowest 10% of ADI neighborhoods (most advantaged) had lower odds (odds ratio, 0.04; 95% CI, 0.00-0.81) of living within 3 miles of a CPP as compared with populations living in the highest 10% of ADI neighborhoods (most disadvantaged).

**Conclusions and Relevance:**

In this cross-sectional study of US communities’ proximity to CPP, we found that neighborhoods potentially exposed to CPP’s hazardous environmental toxicants were more likely to be high ADI (ie, lower socioeconomic status) than low ADI (ie, higher socioeconomic status), highlighting the significantly greater burden of environmental toxicants potentially borne by disadvantaged US populations. Recent federal deregulations are likely to further increase these potential CPP-related toxin exposures. Additional study is needed to determine if these environmental toxicants trigger biological mechanisms underpinning the association between high ADI and poor health.

## Key Points

**Question:**

How do US neighborhoods most exposed to coal-fired power plants (CPP) differ by Area Deprivation Index (ADI), a measure of the adverse social exposome?

**Findings:**

In this cross-sectional study of the US population, individuals living near CPP were more likely to be living in the highest ADI neighborhoods as compared with the lowest ADI neighborhoods. Compared with the US population, these individuals were more likely to be American Indian and/or Alaska Native and White and non-Hispanic.

**Meaning:**

These findings suggest neighborhoods closest to CPP have a disproportionately high ADI.

## Teaser

This cross-sectional study characterizes the populations and adverse social exposome of communities neighboring operational coal-fired power plants.

## Introduction

Health is not distributed equally and is dictated by complex interactions between an individual’s genes and their environment. Although one’s genes are nonmodifiable, environmental exposures (the exposome) can be. Adverse exposome exposures take many forms and can be classified by domain: social, physical, chemical, and biological. The adverse social exposome, as quantified by high Area Deprivation Index (ADI)^1^ scores, has been associated with a multitude of poorer health outcomes. Toxin exposures may be one mechanism through which the adverse social exposome impacts health. Assessments of proximity-based potential toxin exposures through the lens of the adverse social exposome have been limited in the US but may inform mechanistic-focused interventions designed to mitigate health disparities.

Some neighborhoods are less healthy. Individuals living in historically redlined neighborhoods, which are often high ADI, experience poorer health with increased risks of diabetes, hypertension, heart disease, and earlier mortality.^2,3^ These neighborhoods also face higher burdens of toxic air pollution (ie, nitrogen dioxide sulfur dioxide_,_ and fine particulate matter) in part due to disparate sitings of and emissions from coal-fired power plants (CPP).^4,5,6^ Residential communities neighboring CPP are particularly vulnerable to air pollutants, with residential exposure linked to increased mortality, respiratory disease, and adverse birth outcomes.^7,8,9^ Research has examined racial-, poverty-, redlining-, and emissions-based inequities around power plants, but no work to date has examined CPP and their surrounding communities in the context of ADI. ADI is a scientifically validated composite metric used to quantify the adverse social exposome. Importantly, it is a relevant metric for examining health outcomes and health disparities at a geographically precise level that presents opportunities for targeted interventions.

The February 2026 repeal of the Environmental Protection Agency’s (EPA) Endangerment Finding and Mercury and Air Toxics Standards Amendment modify regulatory standards for hazardous air pollutants such as mercury, fine particulate matter, and nitrogen oxides. These changes mean that US neighborhoods located nearest toCPPs may experience even higher levels of CPP-related toxin exposures than in the past.

The aim of this study is to quantify the adverse social exposome (using ADI) of US neighborhoods in close proximity to CPP locations—locations that are more likely to be exposed to higher concentrations of CPP pollutant levels. This work will support communities by promoting science-driven policy action and by forwarding a deeper investigation of the environmental-biological mechanisms that drive health disparities.

## Methods

### Data Sets

A geographic simulation of all operational CPP in the US was constructed using 2024 CPP data provided by the US Energy Information Administration Form 860 (see eAppendix 1 and eTable 1 in Supplement 1 for details on operational plants). To harmonize all datasets in a time-concordant manner, only those plants operable in 2023 were included in the analysis. CPP were geolocated using ArcGIS pro. Concentric circular buffer zones around each CPP of 3, 10, 25, and 50 miles in radius were used to define potential residential exposure to CPP toxicants.^10^ Buffer zones are based on the EPA’s method for defining power plant neighboring communities and literature detailing CPP pollutant dispersion distances.^10,11,12^ Adverse social exposome was measured at the census block-group level using the 2023 national ADI score from the University of Wisconsin’s Neighborhood Atlas and linked using block-group level gisjoin codes. Population characteristics including sex (male or female), age (<18, 18-64, or ≥65 years), race (American Indian and Alaska Native; Asian; Black; Native Hawaiian and Other Pacific Islander; White; other [some other race alone, 2 or more races; 2 or more races: 2 races including some other race; 2 or more races: 2 races excluding some other race; and 3 or more races]), and ethnicity (Hispanic or Latino or Not Hispanic or Latino) were derived from self-reported data in the 2023 American Community Survey. Population characteristics and block group delineations were downloaded from the IPUMS National Historical Geographic Information System version 21.0.^13^ Block-groups offer the most geo-precise and time-concordant operationalization of the construct of neighborhood in publicly available Census data, representing approximately 1500 people. A sensitivity analysis at the block level was conducted to more precisely define exposed populations (see eAppendix 2 and eTable 2 in Supplement 1). This study was classified as exempt by the University of Wisconsin institutional review board as it is not human participants research and exclusively uses published and publicly available datasets without identifiable linkages. No participant consent was required. We followed Strengthening the Reporting of Observational Studies in Epidemiology (STROBE) reporting guidelines for cross-sectional studies.

### Statistical Analysis

All operational CPP in the US in 2023 were located using latitude and longitude, with buffer zones constructed using each CPP as the precise center of a circular ring of 3-, 10-, 25-, and 50-mile radii. Census block-groups with any degree of intersection with the 3-, 10-, 25-, or 50-mile circular buffer regions around the CPP were considered potentially exposed to CPP toxicants and characterized by their population demographics (sex, age, race, and ethnicity) and ADI. Overlapping block groups were deduplicated such that populations living near multiple plants could not be counted more than once. Given the inherent nonindependence between individuals in the 3-, 10-, 25- and 50-mile buffer zones, buffer regions were not compared 1:1, but rather each buffer was compared with the US population. Area-level population apportionment strategies were not employed. Block-groups were grouped into categories of high ADI, defined as those with ADI scores of 81 to 100 (top 20% adverse exposome) and low ADI, defined as those with ADI scores of 1 to 80 (lowest 80%) consistent with prior methods.^14,15,16,17^ Decile groupings are also described for greater precision of CPP distributions; population counts for US deciles are approximated to be 10% of the US population. Descriptive frequencies and odds ratios were used to examine demographic contrasts between each buffer zone’s residential population and the US population; 95% Wald confidence intervals were calculated for all odds ratios. Statistical significance levels were set a priori at *P* levels of less than .05. Data were analyzed using SAS software version 9.4 (SAS Institute) from March to April 2026.

## Results

The 2023 US population included 335 563 144 people (169 517 541 [50.5%] female, 66 294 296 [19.8%] Hispanic, 269 268 846 [80.2%] not Hispanic or Latino, and 212 002 396 [63.1%] White). There were 222 operational CPP in the US in 2023 (Figure 1). A total of 2 783 004 people (0.83% of the US population) lived within 3 miles of a CPP. Communities within 10, 25, or 50 miles had 15 284 984, 60 862 011, and 137 332 672 people, respectively, representing 4.56%, 18.14%, and 40.93% of US population (Table 1). Figure 2 presents the distribution of populations within each CPP buffer zone by ADI percentile.

**Figure 1.**
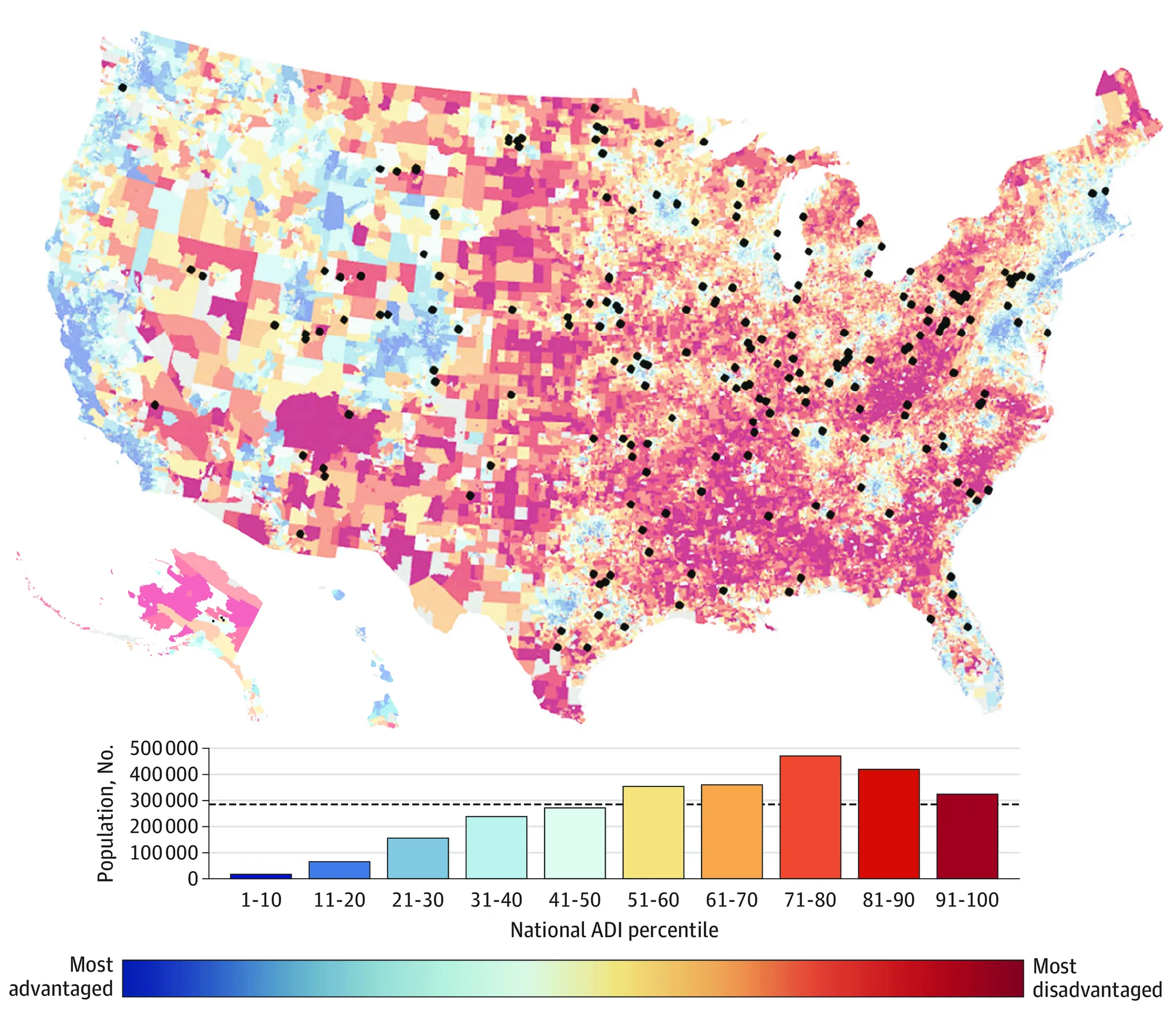
Map and Histogram of Distribution of Coal-Fired Power Plants (CPP) and Adverse Social Exposome Within 3 Miles of a CPP Each black dot represents the location of an operational CPP. Adverse social exposome is operationalized using the national ADI. Darker red areas denote census block groups with the highest levels of adverse social exposome (high ADI). Dark blue areas denote census block groups with the lowest levels of adverse social exposome (low ADI). The histogram at the bottom of the figure illustrates the population distribution of ADI for neighborhoods within 3-miles of a CPP. The black dotted line represents 10% of the total sample population. ADI indicates Area Deprivation Index.

**Table 1.** Demographics Characteristics of the US in 2023 Relative to Distance From an Operational Coal-Fired Power Plant (CPP)

Characteristic	US population, millions (%) (n = 335 563 144)	CPP buffer							
		3 Miles (n = 2 783 004)		10 Miles (n = 15 284 984)		25 Miles (n = 60 862 011)		50 Miles (n = 137 332 672)	
		% of Sample	OR (95% CI)^a^	% of Sample	OR (95% CI)^a^	% of Sample	OR (95% CI)^a^	% of Sample	OR (95% CI)^a^
Sex									
Female	50.52 (169.52)	49.58 (1.38)	1 [Reference]	50.29 (7.69)	1 [Reference]	50.61 (30.80)	1 [Reference]	50.62 (69.52)	1 [Reference]
Male	49.48 (166.05)	50.42 (1.40)	1.04 (0.60-1.18)	49.71 (7.60)	1.01 (0.58-1.76)	49.39 (40.06)	1.00 (0.57-1.73)	49.38 (67.81)	1.00 (0.57-1.73)
Age, y									
<18	22.10 (74.17)	22.10 (74.17)	1 [Reference]	22.32 (3.41)	1 [Reference]	22.48 (13.68)	1 [Reference]	22.27 (30.58)	1 [Reference]
18-64	61.00 (204.69)	61.00 (204.69)	1.01 (0.51-2.02)	60.55 (9.26)	0.98 (0.49-1.95)	60.75 (36.97)	0.98 (0.49-1.94)	60.71 (83.37)	0.99 (0.50-1.96)
≥65	16.90 (56.70)	16.90 (56.70)	1.07 (0.44-2.62)	17.13 (2.62)	1.00 (0.41-2.15)	16.78 (10.21)	0.98 (0.40-2.39)	17.03 (23.38)	1.00 (0.41-2.44)
Race									
American Indian and Alaska Native	0.87 (2.93)	1.37 (0.04)	1.25 (0.08-18.71)	0.91 (0.14)	0.90 (0.05-17.36)	0.71 (0.43)	0.74 (0.03-17.32)	0.67 (.90)	0.70 (0.03-17.24)
Asian	5.77 (19.36)	1.86 (0.05)	0.26 (0.05-1.48)	2.79 (0.43)	0.42 (0.10-1.81)	3.25 (1.98)	0.51 (0.13-2.07)	3.72 (5.11)	0.59 (0.15-2.25)
Black	12.31 (41.29)	7.56 (0.21)	0.49 (0.19-1.28)	11.23 (1.72)	0.79 (0.33-1.89)	14.01 (8.53)	1.03 (0.35-2.39)	14.55 (19.99)	1.07 (0.47-2.47)
Native Hawaiian and Other Pacific Islander	0.19 (0.63)	0.08 (0.002)	0.33 (0.00-1684.86)	0.08 (0.01)	0.36 (0.00-1413.31)	0.09 (0.05)	0.43 (0.00-1204.09)	0.09 (0.12)	0.43 (0.00-1207.04
White	63.18 (212.00)	79.69 (2.22)	1 [Reference]	73.32 (11.21)	1 [Reference]	69.67 (42.40)	1 [Reference]	69.5 (94.07)	1 [Reference]
Other^b^	28.58 (95.91)	16.46 (0.10)	0.46 (0.23-1.29)	19.71 (3.01)	0.59 (0.25-1.18)	20.79 (12.66)	0.66 (0.34-1.28)	20.82 (28.59)	0.66 (0.34-1.28)
Ethnicity									
Hispanic or Latino	19.76 (66.29)	8.41 (0.23)	0.37 (0.16-0.88)	11.69 (1.79)	0.54 (0.25-1.18)	12.23 (7.45)	0.57 (0.26-1.23)	12.49 (17.15)	0.58 (0.27-1.25)
Not Hispanic or Latino	80.24 (269.27)	91.59 (2.55)	1 [Reference]	88.30 (13.50)	1 [Reference]	87.76 (53.41)	1 [Reference]	87.51 (120.18)	1 [Reference]
ADI^c^									
No.	335 563 144^d^	2 699 927^d^	NA	14 952 445^d^	NA	59 695 636^d^	NA	134 621 194^d^	NA
1-80	80	72.30 (1.95)	1 [Reference]	77.66 (11.61)	1 [Reference]	80.16 (47.85)	1 [Reference]	80.62 (108.53)	1 [Reference]
81-100	20	27.70 (0.75)	1.53 (0.79-2.96)	22.34 (3.34)	1.15 (0.58-2.27)	19.84 (11.84)	0.99 (0.49-1.98)	19.38 (26.09)	0.96 (0.48-1.93)

Abbreviations: ADI, Area Deprivation Index; NA, not applicable; OR, odds ratio.

^a^ Odds ratios are purely descriptive as a means of demonstrating magnitudes of difference between categories of buffer zones as compared with the US population. No population sampling is done as this is population-representative data. The ADI and American Community Survey population-level data come with inherent uncertainty. Ninety-five percent Wald confidence intervals were calculated for each odds ratio.

^b^ Other category is comprised of the following American Community Survey Categories: some other race alone; 2 or more races; 2 or more races: 2 races including some other race; 2 or more races: 2 races excluding some other race; and 3 or more races.

^c^ Adverse social exposome is operationalized using the national ADI.

^d^ Total population totals in each of the buffer zones vary as compared with the demographic section of Table 1 because ADI values cannot be defined for every census block group. This is a function of annual ADI calculations, where some block groups are incalculable for a variety of reasons (ie, low population, high group quarters with no American Community Survey data collected, or questionable data integrity as part of the American Community Survey). Please see the Neighborhood Atlas FAQs for detailed ADI construction methods.

**Figure 2.**
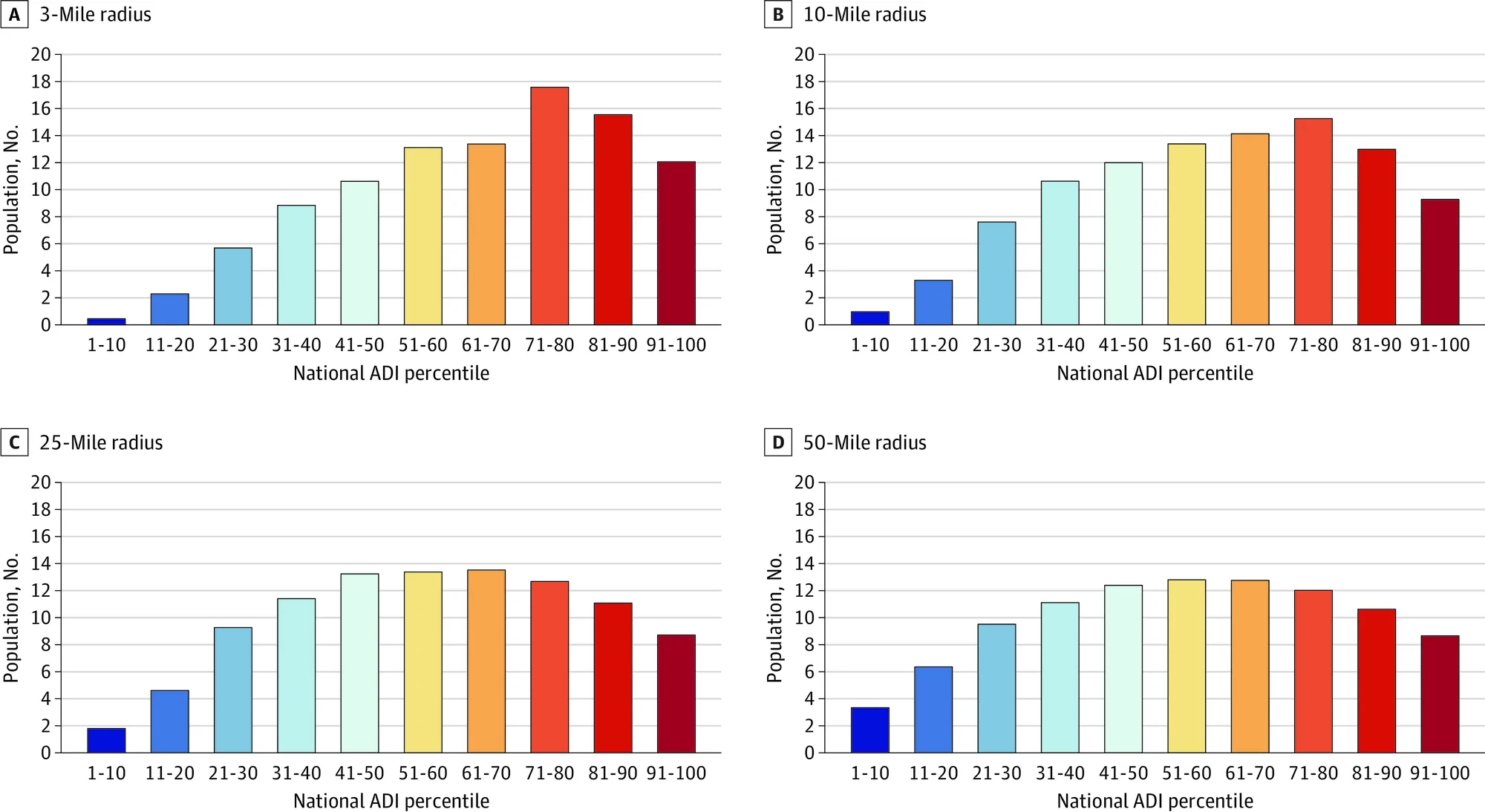
Bar Charts of Distribution of US Population Surrounding Coal-Fired Power Plants by Adverse Social Exposome Distributions of US populations are shown within a 3-mile (A), 10-mile (B), 25-mile (C), and 50-mile (D) radius of the nearest coal-fired power plant. Adverse social exposome is operationalized using the national ADI. Darker red areas denote census block groups with the highest levels of adverse social exposome (high ADI). Dark blue areas denote census block groups with the lowest levels of adverse social exposome (low ADI). ADI indicates Area Deprivation Index.

Individuals living in the highest ADI neighborhoods were more likely to be living within 3 miles of a CPP as compared with those living in the lowest ADI neighborhoods using the US population as a reference (Table 2). Of the 2 783 004 people living within 3 miles of a CPP, 27.70% were in high ADI neighborhoods (most disadvantaged 20%). These populations were more likely to be American Indian and Alaska Native, White, and non-Hispanic (Table 1).

**Table 2.** Distribution of Populations With a 3-, 10-, 25-, and 50-Mile Radius of a Coal-Fired Power Plant (CPP) by Adverse Social Exposome

ADI^b^	CPP buffer							
	3-Mile (n = 2 699 927)^a^		10-Mile (n = 14 952 445)^a^		25-Mile (n = 59 695 636)^a^		50-Mile (n = 134 621 194)^a^	
	% of Sample	OR (95% CI)	% of Sample	OR (95% CI)	% of Sample	OR (95% CI)	% of Sample	OR (95% CI)
1-10	0.48 (0.01)	1 [Reference]	0.96 (0.14)	1 [Reference]	1.82 (1.09)	1 [Reference]	3.40 (4.57)	1 [Reference]
11-20	2.33 (0.06)	4.85 (0.19-122.47)	3.36 (0.50)	3.50 (0.31-39.83)	4.64 (2.77)	2.55 (0.37-17.48)	6.40 (8.62)	1.88 (0.39-9.14)
21-30	5.73 (0.15)	11.94 (0.55-257.88)	7.67	7.99 (0.80-79-36)	9.32 (5.56)	5.12 (0.83-31.39)	9.54 (12.85)	2.81 (0.62-12.79)
31-40	8.88 (0.24)	18.52 (0.89-384.78)	(1.15)	11.13 (1.16-107.13)	11.44 (6.83)	6.29 (1.05-37.76)	11.14 (14.99)	3.28 (0.73-14.65)
41-50	10.66 (0.29)	22.21 (1.08-455.95)	10.68	12.57 (1.32-119.97)	13.29 (7.93)	7.30 (1.23-43.30)	12.43 (16.74)	3.66 (0.83-16.15)
51-60	13.17 (0.36)	27.44 (1.35-556.94)	(1.60)	13.99 (1.48-132.54)	13.40 (8.00)	7.36 (1.24-43.63)	12.85 (17.30)	3.78 (0.86-16.64)
61-70	13.43 (0.36)	27.98 (1.38-567.40)	12.07 (1.81)	14.77 (1.56-139.47)	13.55 (8.09)	7.45 (1.25-44.08)	12.80 (17.23)	3.76 (0.85-16.58)
71-80	17.62 (0.48)	36.71 (1.83-736.04)	13.43 (2.01)	15.96 (1.70-150.01)	12.70 (7.58)	6.98 (1.17-41.53)	12.06 (16.23)	3.55 (0.80-15.72)
81-90	15.59 (0.42)	32.48 (1.61-654.33)	14.18 (2.12)	13.58 (1.43-128.93)	11.09 (6.62)	6.09 (1.01-36.71)	10.67 (14.37)	3.14 (0.70-14.10)
91-100	12.11 (0.33)	25.23 (1.24-514.28)	15.32 (2.29)	9.70 (1.00-94.49)	8.75 (5.22)	4.81 (0.78-29.72)	8.71 (11.72)	2.56 (0.56-11.82)

Abbreviations: ADI, Area Deprivation Index; OR, odds ratio.

^a^ Total population in each of the buffer zones vary as compared with Table 1 because ADI values cannot be defined for every census block group. This is a function of annual ADI calculations, where some block groups are incalculable for a variety of reasons (ie, low population, high group quarters with no American Community Survey data collected, or questionable data integrity as part of the American Community Survey). Please see the Neighborhood Atlas FAQs for detailed ADI construction methods.

^b^ Adverse social exposome is operationalized using the national ADI.

CPP were rarely located in the lowest 10% of ADI percentiles (most advantaged) neighborhoods. Less than 3.5% of the sample population in the 3-, 10-, 25-, and 50-mile buffer zones were in the most advantaged ADI neighborhoods (0.48%, 0.96%, 1.82%, and 3.40%, respectively) (Figure 2). Populations living in lowest 10% of ADI neighborhoods (most advantaged) had lower odds (odds ratio, 0.04; 95% CI, 0.00-0.81) of living within 3 miles of a CPP as compared with populations living in the highest 10% of ADI neighborhoods (most disadvantaged).

## Discussion

In this geosimulation study, neighborhoods closest to CPPs were disproportionately high ADI. It is well-established that populations living in adverse social exposomes indexed by high ADI experience worse health outcomes,^18,19,20^ and the location of CPPs may exacerbate this burden. Previous work has demonstrated that communities in closer proximity to power plants faced greater burdens of toxic air pollution and simultaneously experience worse health outcomes across the life-course. With an estimated 1% to 18% of the US population living near to CPP (within 3, 10, or 25 miles), there is tremendous opportunity to mitigate residential exposure to proximity-based potential hazardous toxicants and improve community health.

Notably, this study found that CPPs are rarely located near low ADI neighborhoods, which are typically wealthy and well-resourced. When examined by dichotomized groupings (80% least disadvantaged vs 20% most disadvantaged), we saw that more disadvantaged census block groups are more likely to be located within the 3- and 10- mile circular buffers, but this did not reach statistical significance. However, when examining the deciles separately, the picture becomes more nuanced. It is still the more disadvantaged census block groups that appear more likely to be located within 3, 10, 25, or 50 miles of a CPP, but the groups most likely to experience consequences are those spanning the fourth to ninth deciles of ADI. This is a striking pattern that may reflect differential influence in public decision-making processes for those residing in low vs high ADI neighborhoods. However, given the cross-sectional nature of this work, additional study is required to understand the temporality of coal-fired power plant sitings and neighborhood characteristics.

Studies have repeatedly demonstrated the negative health impacts of CPP exposure.^7,8,9^ The recent repeals of the Environmental Protection Agency’s (EPA) Endangerment Finding and 2024 amendments to the Mercury and Air Toxics Standards Amendment may serve to compound the known existing harms. These deregulation efforts are anticipated to result in increased population exposure to hazardous heavy metals and particulate matter in CPP air pollution, particularly harmful for those living nearest to CPP who reside in disproportionately high ADI neighborhoods. Given the known burden of poor health in high ADI neighborhoods, this additional potential toxin exposure may further compound existing health disparities.

This study used the most up-to-date population-level data for demographics, ADI, and operational coal-fired power plant locations, making it broadly generalizable and timely. Yet, there are limitations. This geosimulation takes a cross-sectional approach. Accounting for life-course exposure profiles is important and should be included in future research. We did not incorporate CPP capacity factor or wind patterns into our analysis but created multiple circular buffer zones consistent with previous literature to account for some degree of differential pollutant exposure. We used circular buffer zones with any degree of overlap with census block groups without incorporating area-based population apportionment strategies or direct air pollution measurements. This method may introduce a degree of exposure misclassification, yet at a population level, this method provides a reasonable and policy-actionable approach to mitigate community-level harm. Given the use of multiple buffer zones, the overestimation of exposed populations will be most likely within the 3-mile buffer zone. Overlapping buffers were deduplicated, such that populations were not counted more than once. In doing this, our analysis cannot quantify those populations with more severe potential exposures (ie, those living in close proximity to multiple CPP). For comparative purposes, US ADI decile population counts were assumed and approximated to be 10% of the 2023 US population.

## Conclusions

In this cross-sectional observational study examining US communities’ proximity to CPP, we found that CPPs were more often located in high ADI neighborhoods, resulting in populations with greater adverse exposome burden experiencing the highest potential exposures to CPP toxicants. Loss of environmental protection standards for clean air may disproportionately impact high ADI communities.

## Notes

Supplement 1. eAppendix 1. Description of Operational Coal-Fired Power Plants eTable 1. Coal-Fired Power Generator Status Code Description eAppendix 2. Sensitivity Analysis eTable 2. Distribution of Population Within 3 Miles of a Coal-Fired Power Plant Using Census Blocks

Supplement 2. Data Sharing Statement
