# Supplementary material for: Distribution of Coal Power, Pollution, and Poverty
Source: JAMA Netw Open. 2026 Nov 5;9(11):e2642335. doi: 10.1001/jamanetworkopen.2026.42335 (PMC13741158; doi:10.1001/jamanetworkopen.2026.42335)
Supplement: Supplementary file 1 [file jamanetwopen-e2642335-s001.pdf]

Supplemental Online Content

Melcher EM, Buckingham WR, Keller SA, Kind AJH. Distribution of coal power, pollution, and poverty. *JAMA Netw Open*. 2026;9(11):e2642335. doi:10.1001/jamanetworkopen.2026.42335

eAppendix 1. Description of Operational Coal-Fired Power Plants

eTable 1. Coal-Fired Power Generator Status Code Description

eAppendix 2. Sensitivity Analysis

eTable 2. Distribution of Population Within 3 Miles of a Coal-Fired Power Plant Using Census Blocks

This supplemental material has been provided by the authors to give readers additional information about their work.

© 2026 Melcher EM et al. *JAMA Network Open*.

eAppendix 1. Description of Operational Coal-Fired Power Plants

1. In the Energy Information Administration (EIA) Form 860 data set coal-fired power generators can be marked as:

eTable 1. Coal-Fired Power Generator Status Code Description

Generator Status Code	Generator Status Code Description (EIA definition)	Melcher et. al. Classification
Retired (RE)	No longer in service and not expected to be returned to service	Non-Operational
Cancelled (CN)	Cancelled. Previously reported as planned.	Non-Operational
Indefinitely Postponed (IP)	Planned new indefinitely postponed, or no longer in resource plan.	Non-Operational
Operational (OP)	In service (commercial operation) and producing some electricity. Includes peaking units that are run on as needed (intermittent or seasonal) basis)	Operational
Out of Service (OS)	Was not used for some or all of the reporting period and is NOT expected to be returned to service in the next calendar year.	Operational
Out of Service (OA)	Was not used for some or all of the reporting period but is expected to be returned to service in the next calendar year.	Operational
Standby/Backup (SB)	Available for service but not normally used (has little or no generation during the year) for this reporting period.	Operational

Note: Generator status code descriptions are derived directly from Form EIA-860 Instructions for the Annual Electric Generator Report and reference tables 4-6 in the LayoutY2024 Excel document.

2. Individual generating units in the data set *not* marked as operational (OP):
    a. 4 are OA
    b. 13 are OS
    c. 4 are SB
    d. 12 marked as RE, but all of their planned retirement dates are after Jan 2024, meaning they were still operational in 2023.
3. Plants with non "OP" generating units and operational classification during analysis year 2023:
    a. Plant code 136: This plant had one generator marked as retiring in 2024, but its other generator has no planned retirement. This generator was still operational during analysis year 2023.
    b. Plant code 963: This plant had one generator marked as retiring in 2024, but its other generator has no planned retirement. This generator was still operational during analysis year 2023.
    c. Plant code 1167: This plant had one generator marked as retiring in 2024, but its other generator has no planned retirement. This generator was still operational during analysis year 2023.

© 2026 Melcher EM et al. *JAMA Network Open*.

d. Plant code 1364: This plant had one generator marked as retiring in 2024, but its other generator has no planned retirement. This generator was still operational during analysis year 2023.
e. Plant code: 2367: Both generators at this plant are marked as OS. No other CPP generators exist at this plant. It was considered operational during analysis year 2023 per Table in point #1 above.
f. Plant code 2718: Both generators at this plant were marked for retirement in 2024, but remained operational during the analysis year 2023.
g. Plant code 2914: One generator at this plant is marked as SB, but the other is operational. This plant was operational during 2023.
h. Plant code 2935: Two generators at this plant are marked as OS, but the other two generators at this plant are OP, so this plant is considered operational during analysis year 2023
i. Plant code 2936: Two generators at this plant are marked as SB, but the other two generators are operational, so this plant is operational during analysis year 2023
j. Plant code 3122: There is only one generator at this plant, and it is marked for retirement in 2024, but it remains operational during analysis year 2023.
k. Plant code 4041: Two out of four generators were marked for retirement in 2024, but both were still operational during analysis year 2023.
l. Plant code: 6155: Two of two generators were marked for retirement in 2024, but both remained operational during analysis year 2023.
m. Plant code 6705: One of four generators at this plant was marked as OA, but the remaining 3 are OP. This plant was operational during analysis year 2023.
n. Plant code 10234: One of two generators at this plant was marked as OA, but the other was marked as OP. This plant was operational during analysis year 2023.
o. Plant code 10678: There was only one generator at this plant, and it was marked as retiring in 2024. This plant remained operational during analysis year 2023.
p. Plant code 10684: One of three generators at this plant was marked as OS while the other two were OP. This plant was operational during 2023.
q. Plant code 10743: There was only one generator at this plant and it was marked as OS. This plant was operational during 2023.
r. Plant code 10849: Two of two generators at this plant were marked as OS. This plant was operational during 2023.
s. Plant code 50711: Three of four generators at this plant were marked OS, while one generator was marked as OP. This plant was operational during 2023.
t. Plant code 50933: This plant only had one generator which was marked as OS. This plant was operational during 2023.
u. Plant code 54867: This plant only had one generator which was marked as SB. This plant was operational during 2023.
v. Plant code 47915: Two of six generators at this plant were marked as OA. The remaining four were marked as OP. This plant was operational during 2023.
w. Plant code 57949: This plant contained two generators, one was marked as OS and the other was marked as OP. This plant was operational during 2023.

© 2026 Melcher EM et al. *JAMA Network Open*.

eAppendix 2. Sensitivity Analysis

The main analyses in this manuscript examined the populations in regions of overlap between the 3-, 10-, 25-, and 50-miles buffer regions around operational coal-fired power plants (CPP) and census block groups in the United States. The areas of overlap, examined as proximity-based proxies for potential exposure to CPP pollutants, were characterized by population counts, demographic characteristics, and area deprivation index scores. Census block-groups with any degree of intersection with the 3-, 10-, 25, or 50-mile circular buffer regions around the CPP were included in the resulting potentially exposed populations. No area-based population apportionment strategies were utilized, which may have led to overestimations of exposed populations (i.e., population misclassification), particularly at 3-mile CPP buffer intersection region. As such, a sensitivity analysis was performed at a more precise geography in order to minimize the risk of population misclassification. In this sensitivity analysis, all operational CPP in the US in 2023 were located using latitude and longitude, with buffer zones constructed using each CPP as the precise center of a circular ring of 3-mile radius. Census blocks, rather than block-groups, with any degree of intersection with the 3-mile circular buffer regions around the CPP were characterized by their population. Overlapping block groups were deduplicated prior to population enumeration. A census block is the smallest geographic unit for which data is provided by the US census bureau. However, population level counts are only provided for the decennial census years, so this analysis used data from the 2020 census. This presents a lack of harmony with the CPP data, which is from the year 2023, however, it is the best proxy measurement for population counts at a more precise geography. Furthermore, the area deprivation index is only available at the census block group level, so it could not be incorporated into this sensitivity analysis. The purpose of this analysis is to instead demonstrate the degree of exposure misclassification in the original analysis.

eTable 2. Distribution of Population Within 3 Miles of a Coal-Fired Power Plant Using Census Blocks

	**3-Mile CPP Buffer** *(N=1,657,897)*
**Percent of 2020 US Population** *(N=331,449,281)*	0.50%
**Percent of 2023 US Population**[b] *(N=335,563,144)*	0.49%

Note: Census block population data is only available for decennial census years (i.e., 2000, 2010, 2020). As such data from the 2020 census was used to approximate our 2023 census block-group calculations in the main analysis.

© 2026 Melcher EM et al. *JAMA Network Open*.
