# Supplementary material for: Distribution of Coal Power, Pollution, and Poverty
Source: JAMA Netw Open. 2026 Nov 5;9(11):e2642335. doi: 10.1001/jamanetworkopen.2026.42335 (PMC13741158; doi:10.1001/jamanetworkopen.2026.42335)
Supplement: Supplementary file 2 [file jamanetwopen-e2642335-s002.pdf]

Data Sharing Statement

Melcher. Distribution of coal power, pollution, and poverty. *JAMA Netw Open*. Published November 5, 2026. doi:10.1001/jamanetworkopen.2026.42335

Data
Additional Information: NA
Data types: Area Deprivation Index: CSV; Block group demographics: CSV; Block group delineations: shp; coal-fired power plant: xlsx
How to access data: CPP data is available via the US Energy Information Administration Form 860. Area Deprivation Index data is available on the University of Wisconsin's Neighborhood Atlas. Geospatial delineations are available from the University of Minnesota's IMPUMS National Historical Geographic Information System version 21.0. All population-level and demographic data gathered by the American Community Survey is available from the University of Minnesota's IMPUMS National Historical Geographic Information System version 21.0.
When available: Currently available

Supporting Documents
Document types: The supplemental file includes (1) a description of operational coal-fired power plants and their generator status codes and (2) a sensitivity analysis for the distribution of populations within 3-miles of a coal-fired power plant using census block groups.

Additional Information
Who can access the data: All data used in these analyses are publicly available.
Types of analyses: Cross-sectional geospatial analysis
Mechanisms of data availability: Online
Any additional restrictions: NA
